# Temperature Affects the Host Range of Rhabdochlamydia porcellionis

**DOI:** 10.1128/aem.00309-23

**Published:** 2023-04-12

**Authors:** Bastian Marquis, Silvia Ardissone, Gilbert Greub

**Affiliations:** a Institute of Microbiology of the University Hospital Center and the University of Lausanne, Lausanne, Switzerland; University of Nebraska-Lincoln

**Keywords:** *Chlamydia*, arthropods, environmental microbiology

## Abstract

The *Rhabdochlamydiaceae* family is a recent addition to the *Chlamydiae* phylum. Its members were discovered in cockroaches and woodlice, but recent metagenomics surveys demonstrated the widespread distribution of this family in the environment. It was, moreover, estimated to be the largest family of the *Chlamydiae* phylum based on the diversity of its 16S rRNA encoding gene. Unlike most Chlamydia-like organisms, no *Rhabdochlamydiaceae* member could be cultivated in amoebae, and its host range remains unknown. We tested the permissivity of various mammalian and arthropod cell lines to determine the host range of Rhabdochlamydia porcellionis, the only cultured representative of this family. While growth could initially be obtained only in the Sf9 cell line, lowering the incubation temperature of the mammalian cells from 37°C to 28°C allowed the growth of *R. porcellionis*. Furthermore, a 6-h exposure to 37°C was sufficient to irreversibly block the replication of *R. porcellionis*, suggesting that this bacterium either lost or never acquired the ability to grow at 37°C. We next sought to determine if temperature would also affect the infectivity of elementary bodies. Although we could not purify enough bacteria to reach a conclusive result for *R. porcellionis,* our experiment showed that the elementary bodies of Chlamydia trachomatis and Waddlia chondrophila lose their infectivity faster at 37°C than at room temperature. Our results demonstrate that members of the *Chlamydiae* phylum adapt to the temperature of their host organism and that this adaptation can in turn restrict their host range.

**IMPORTANCE** The *Rhabdochlamydiaceae* family is part of the *Chlamydiae*, a phylum of bacteria that includes obligate intracellular bacteria sharing the same biphasic developmental cycle. This family has been shown to be highly prevalent in the environment, particularly in freshwater and soil, and despite being estimated to be the largest family in the *Chlamydiae* phylum is only poorly studied. Members of the *Rhabdochlamydiaceae* have been detected in various arthropods like ticks, spiders, cockroaches, and woodlice, but the full host range of this family is currently unknown. In this study, we showed that *R. porcellionis*, the only cultured representative of the *Rhabdochlamydiaceae* family, cannot grow at 37°C and is quickly inactivated at this temperature. A similar temperature sensitivity was also observed for elementary bodies of chlamydial species adapted to mammals. Our work demonstrates that chlamydiae adapt to the temperature of their reservoir, making a jump between species with different body temperatures unlikely.

## INTRODUCTION

The *Chlamydiae* phylum includes obligate intracellular bacteria that share the same biphasic developmental cycle composed of an extracellular infectious stage, the elementary body (EB), and an intracellular replicative form, the reticulate body (1). While historically restricted to the human pathogens of the Chlamydiaceae family (2, 3), this order has seen a rapid expansion during the past decades as new species were discovered in the environment (4–12). Estimations based on sequence diversity in the 16S rRNA encoding gene predict hundreds of unknown family-level lineages (5). It is now evident that far from being restricted to mammals, chlamydiae are highly prevalent in the environment and successfully adapted to different ecological niches and host organisms (5, 13–15).

Since they diverged from their common ancestor hundreds of millions of years ago (3, 16), the different families of the *Chlamydiae* phylum specialized for specific hosts, often losing the ability to infect other organisms in the process. For instance, species of the Chlamydiaceae family, while highly adapted to vertebrates, are seemingly unable to replicate in amoebae (17–19). Conversely, members of the *Parachlamydiaceae* family grow efficiently in amoebae but poorly, if at all, in mammalian and insect cell lines, likely due to their inability to inhibit apoptosis (20–24). Some other families, like the *Simkaniaceae* and *Waddliaceae* families, seem to have conserved wider host ranges, as cultured representatives can grow in a wide variety of cell types (19, 21, 25–27). It is, however, unclear how the *in vitro* host range of these bacteria translates *in vivo*, as immortalized cell lines grown in axenic medium in the absence of an immune system are far from the conditions expected in most multicellular organisms. The host range of some chlamydiae is thus well determined, while it remains unknown for others.

The picture is clearer for the *Rhabdochlamydiaceae* family, a recent addition to the *Chlamydiae* phylum. Members of this family were initially discovered in cockroaches (28) and woodlice (29) and later detected in ticks and spiders (30–34), while a distant relative was recently identified in the amoeba Dictyostelium discoideum (35). Interestingly, *Rhabdochlamydiaceae* were also detected in patients suffering from respiratory infections (36–38) or inflammatory skin disorders (39), suggesting a potential pathogenic role of these bacteria. Despite being hypothesized to be the most diverse clade of the *Chlamydiae* phylum (5), the *Rhabdochlamydiaceae* family is poorly studied, and only one species, isolated from the rough woodlouse, has been cultured so far (40). Similarly to Chlamydia trachomatis, Rhabdochlamydia porcellionis was shown to inhibit apoptosis and could not be cultured in amoebae (40), hinting at a specialization for multicellular organisms. In line with this hypothesis, several arthropod cell lines were shown to sustain the growth of *R. porcellionis* (40). Unlike the Chlamydiaceae, *Rhabdochlamydiaceae* were associated not with animal hosts but with soil and freshwater environments (13), and while various arthropods were demonstrated to be a reservoir for *Rhabdochlamydiaceae*, their full host range is still unknown.

In this study, we sought to study the host range of *R. porcellionis*. We tested the permissivity of mammalian and arthropod cell lines to *R. porcellionis* using immunofluorescence, electron microscopy, and quantitative PCR (qPCR). We could demonstrate that *R. porcellionis* is unable to withstand short exposures to 37°C and cannot grow at 33 and 37°C. Mammalian cells were, however, permissive to these bacteria when incubated at 28°C. *R. porcellionis* thus appears to have adapted to the temperature of its host and to have lost the ability to infect organisms with a higher body temperature in the process.

## RESULTS

### *R. porcellionis* has a limited host range and a long replication cycle.

To study the host range of *R. porcellionis* in mammalian cells, we tested the permissivity of pneumocytes (A549), endometrial cells (Ishikawa), and fibroblasts (McCoy). The first two cell lines are indeed known to be permissive to different Chlamydia-like organisms (25, 27), while McCoy cells are frequently used to propagate members of the Chlamydiaceae family. In addition to the Sf9 cell line, already used for the subculture of *R. porcellionis* (40), we tested a tick cell line (IRE/CTVM19) and a mosquito cell line (C6/36), as those were derived from organisms likely closer to the natural reservoir of the *Rhabdochlamydiaceae* family than mammalian cell lines.

**FIG 1:**
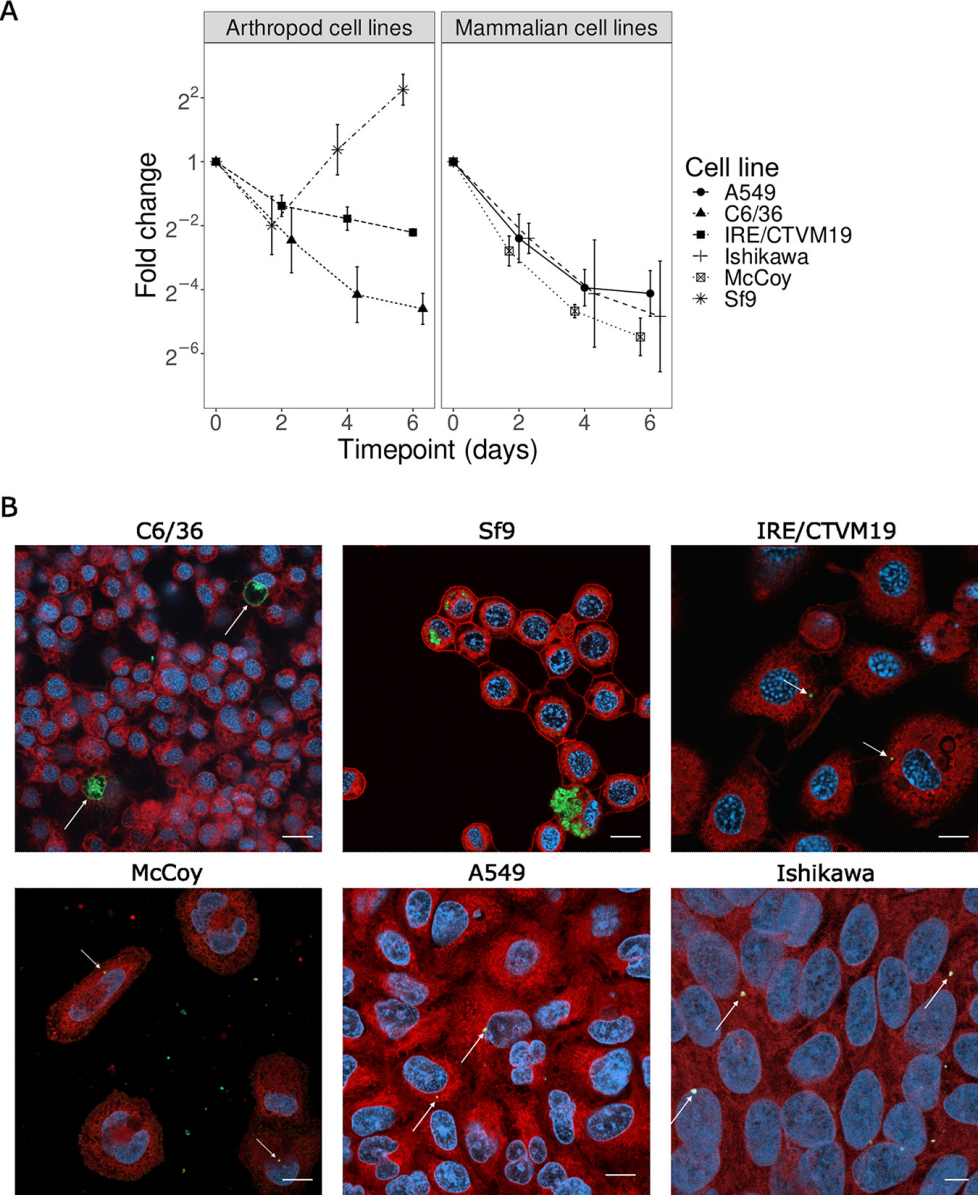
Permissivity of arthropod and mammalian cell lines to *R. porcellionis.* (A) The *y* axis represents the fold change of the number of genome copies per microliter relative to the initial time point. The results are shown as the mean and standard deviation from three biological replicates. (B) *R. porcellionis* in mammalian and arthropod cell lines at 6 days postinfection. Growth could be observed only in Sf9 cells. The reticulate bodies do not appear to be grouped in an inclusion and seem to be replicating directly in the cytoplasm. The enlarged bodies in the C6/36 cell line are likely aberrant bodies. Bacteria appear to have been internalized in all the other cell lines but failed to replicate. White arrows indicate enlarged bacteria in C6/36 cells and internalized EBs in the other cell lines. Cells were stained with concanavalin A (red), DAPI (blue), and anti-*Simkania* antibody (green); bar, 10 μm.

**FIG 2:**
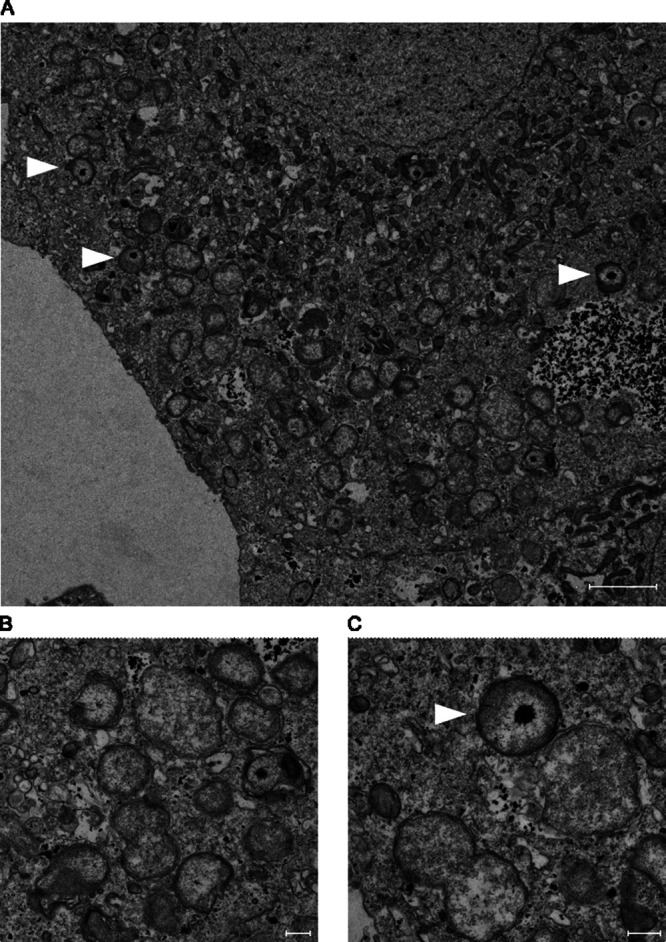
Transmission electron micrographs of *R. porcellionis* in Sf9 cells at 6 days postinfection. (A to C) Infected Sf9 cells harboring numerous bacteria. Reticulate bodies, some of which are undergoing binary fission, can be observed along with intermediate bodies with condensed DNA. The bacteria do not appear to be grouped in an inclusion. Sf9 cells were infected with *R. porcellionis* at an MOI of ~1. White arrowheads, intermediate bodies. Bar, 2 μm (A) or 200 nm (B and C).

Growth could be observed in Sf9 cells (Fig. 1A), with a doubling time of 20.4 h (standard deviation [SD] = 1.9 h), comparable to that of Simkania negevensis (21 h) but longer than that of Waddlia chondrophila (4 h) in the same cell line (21, 25). Immunofluorescence showed heavily infected Sf9 cells at 6 days postinfection (Fig. 1B); however, reticulate bodies appeared disseminated in the cytoplasm of the host cell and did not seem to be enclosed in an inclusion (Fig. 1B). This observation was confirmed in electron microscopy micrographs, where bacteria appear to replicate in the cytoplasm of the host cell without any visible inclusion (Fig. 2A to C). Interestingly, unlike *S. negevensis*, *R. porcellionis* could not grow in mammalian cells and, more surprisingly, failed to grow in all arthropod cell lines except Sf9 cells (Fig. 1A). While bacteria were internalized in all cells tested (Fig. 1B), they either failed to initiate replication or formed aberrant bodies measuring more than 5 μm (in C6/36 cells).

### *R. porcellionis* is unable to replicate at 37°C.

**FIG 3:**
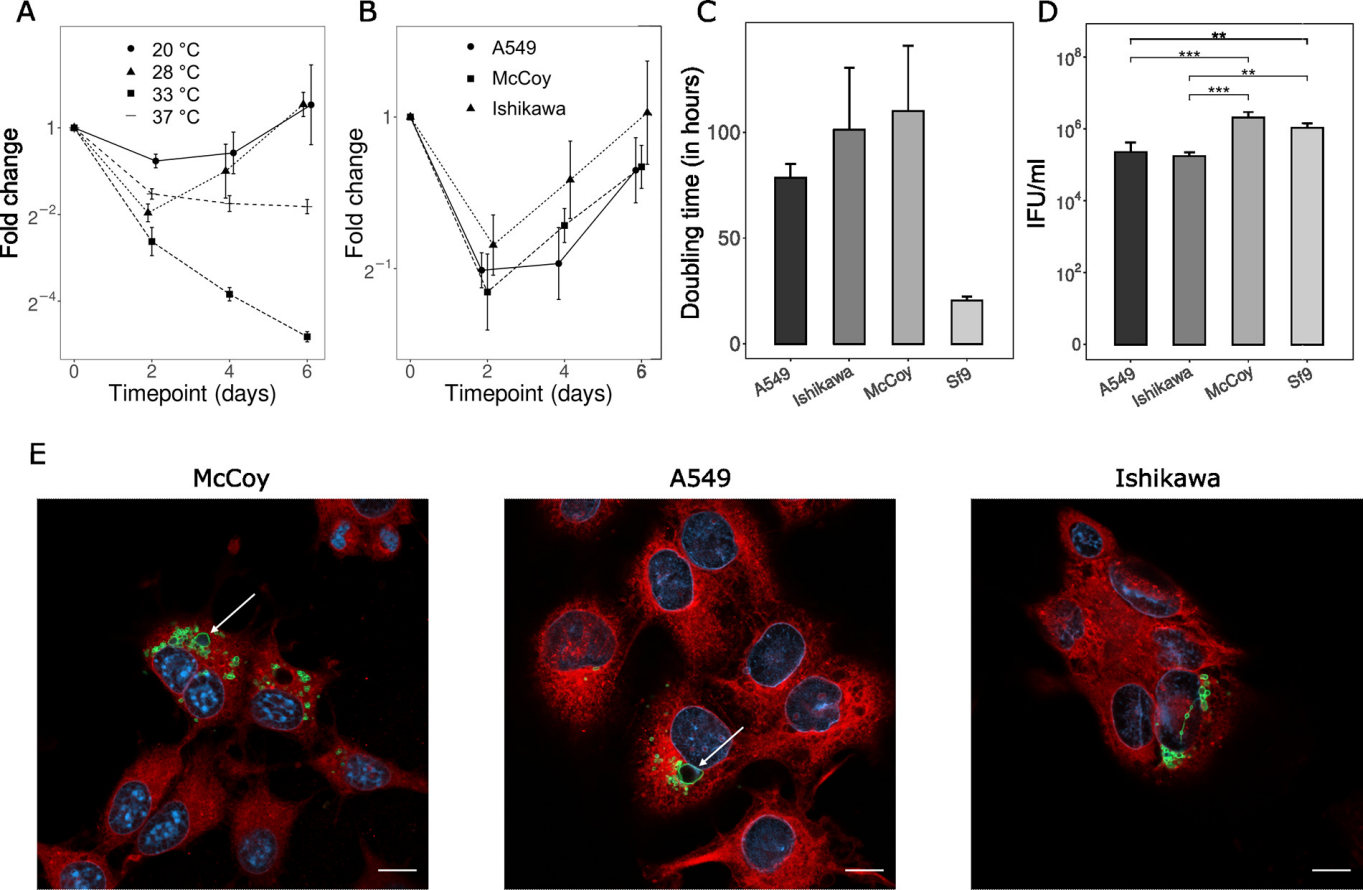
The growth of *R. porcellionis* in mammalian cells depends on temperature. (A) Growth kinetics of *R. porcellionis* in Sf9 cells incubated at 20, 28, 33, and 37°C. (B) Growth kinetics of *R. porcellionis* in mammalian cells incubated at 28°C. In both panel A and panel B, the *y* axis represents the fold change relative to the initial time point. (C) Doubling time of *R. porcellionis* in the different cell lines. Doubling times were estimated by dividing 48 h by the log_2_ of the highest fold change observed between two consecutive time points. Despite the marked difference between the doubling time in Sf9 and the other cell lines, the Kruskal-Wallis test was not statistically significant (*P* = 0.06). (D) IFU count of *R. porcellionis* grown in different cell lines at 28°C. The cells were fixed at 6 days postinfection. The tendency of infected Sf9 cells to detach from the glass coverslips could induce an underestimation of the IFU count. A one-way ANOVA revealed that there was a statistically significant difference between at least two cell lines (*P* value = 0.0002). The plot shows the results of the Tukey honestly significant difference test for the pairwise comparison of the IFU count in the different cell lines (**, <0.01; ***, <0.001). (E) McCoy, A549, and Ishikawa cells infected with *R. porcellionis*, incubated at 28°C, and fixed at 6 days postinfection. The two enlarged bodies in McCoy and A549 cells are likely aberrant bodies (white arrows). Cells were stained with concanavalin A (red), DAPI (blue), and anti-*Simkania* antibodies (green). Bar, 10 μm. The results show the mean and standard deviation from three biological replicates.

*Rhabdochlamydiaceae* have been detected in various arthropods such as ticks (34, 41), spiders (42), cockroaches (28), and woodlice (29). As those organisms are poikilothermic and have a lower body temperature than mammals (43, 44), we reasoned that the *Rhabdochlamydiaceae* family might have adapted to the lower temperature of its host organisms and either lost or never acquired the ability to grow at 37°C. To test this hypothesis, we infected Sf9 cells with *R. porcellionis* and incubated them at 20, 28, 33, and 37°C. *R. porcellionis* grew at 20 and 28°C but not at 33 or 37°C (Fig. 3A). The lack of growth at 33 or 37°C could, however, be due to the loss of permissivity of Sf9 cells at those temperatures. As a control for this, we infected Sf9 cells with W. chondrophila, another chlamydia-like organism known to have a wide host range and to grow at both 28 and 37°C (21, 26, 27), and incubated them at 28 and 37°C. Unlike *R. porcellionis*, W. chondrophila replicated at both temperatures (see Fig. S1 in the supplemental material), suggesting that Sf9 cells retain their permissivity at 37°C. This, however, does not exclude the possibility of Sf9 cells selectively losing their permissivity to *R. porcellionis* at 37°C.

To test if the absence of growth in mammalian cells was also an effect of temperature, we infected A549, Ishikawa, and McCoy cells with *R. porcellionis* and lowered the incubation temperature to 28°C. This change indeed allowed the growth of *R. porcellionis* (Fig. 3B) in mammalian cells, although the doubling time appeared to be longer and more variable than that in Sf9 cells (Fig. 3C). The long doubling time (Fig. 3C), the absence of intermediate bodies, and the distorted appearance of the bacteria in McCoy cells (Fig. S2) indicate that those cells might be too different from the natural host of *R. porcellionis* to allow efficient growth.

We finally compared the infection efficiencies of *R. porcellionis* in A549, McCoy, Ishikawa, and Sf9 cells by measuring the inclusion-forming unit (IFU) count at 6 days postinfection. As shown in Fig. 3D, the IFU count was significantly lower in Ishikawa and A549 cells than in Sf9 cells. Surprisingly, there was no difference between McCoy and Sf9 cells. This might, however, be due to an underestimation of the IFU count in the latter, as infected Sf9 cells tend to detach from glass coverslips.

### Transient exposure to 37°C irremediably blocks the replication of *R. porcellionis*.

**FIG 4:**
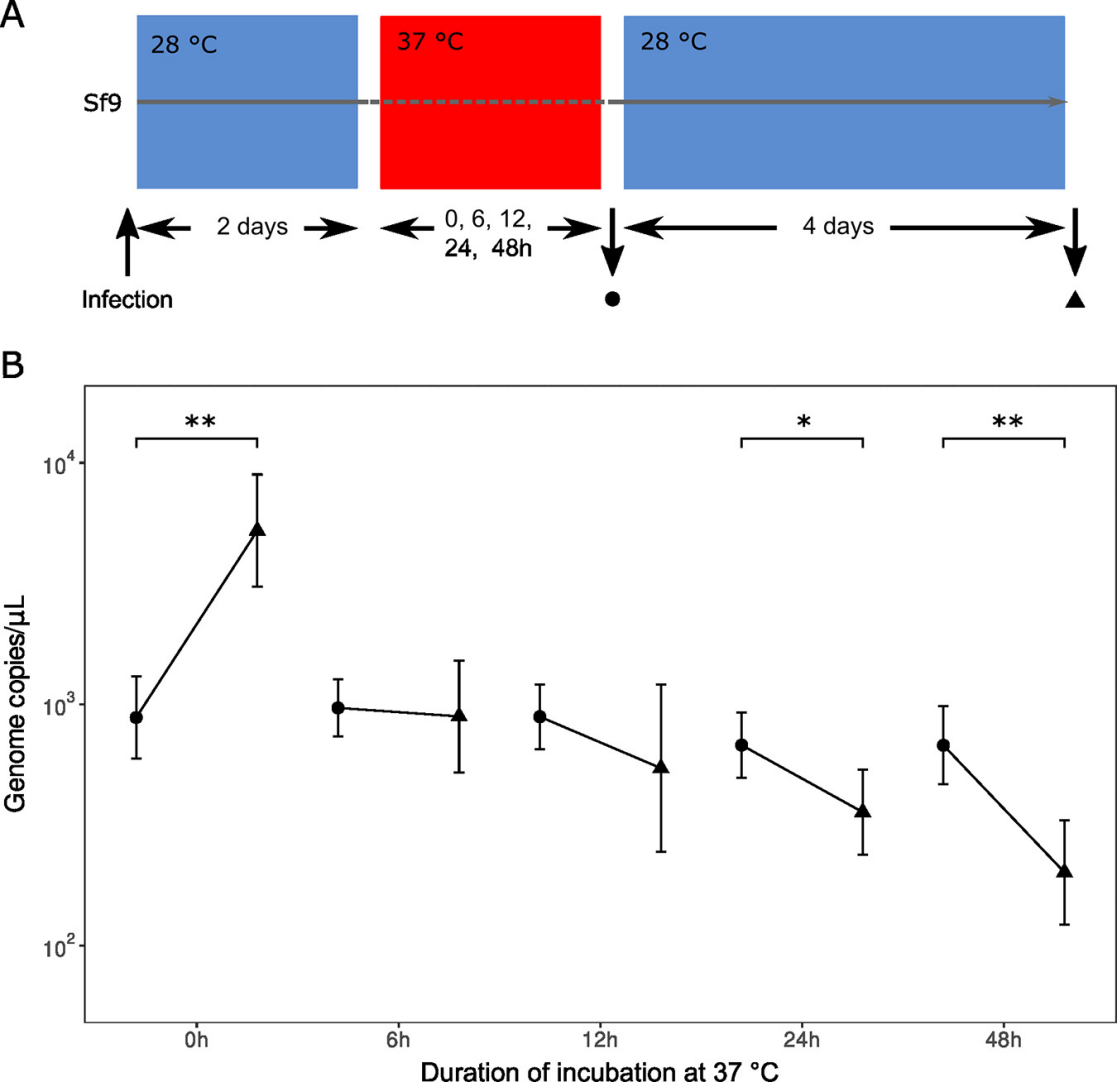
Effect of a transient exposure to 37°C on the replication of *R. porcellionis* in Sf9 cells. (A) Summary of the experimental design. (B) The graph shows the number of bacteria (genome copies/microliter) immediately after the exposure to 37°C (circles) and after 4 days of recovery at 28°C (triangles). The comparison between the two time points was made with a paired *t* test and corrected for multiple testing with the Holm stepdown procedure. The results show the mean and standard deviation from three biological replicates (*, <0.05; **, <0.001).

Members of the *Chlamydiae* are known to enter a third nonreplicative stage, the aberrant body, when exposed to stresses such as antibiotic exposure (45), nutrient deprivation (46), or heat shock (47). Aberrant bodies are typically described as nonreplicating enlarged cells able to resume their regular cycle once the stress disappears, although their morphology was shown to vary as a function of the stresses (48, 49). We thus wondered whether *R. porcellionis* could similarly recover and resume its growth cycle after an exposure to 37°C. To determine this, Sf9 cells at 2 days postinfection were incubated at 37°C for various durations. The infected cells were then further incubated at 28°C for four additional days to check for bacterial growth after stress removal. The growth was assessed by measuring the number of genome copies at the end of the incubation at 37°C and after 4 days of recovery at 28°C (Fig. 4A).

Our experiment shows that a transient exposure to 37°C as short as 6 h irreversibly blocks the replication of *R. porcellionis* (Fig. 4B). The effect of the temperature shift seems to be more deleterious for longer incubation at 37°C, as expected. However, the complete experiment lasted from 6 days, for the samples subjected to the 6-h shift at 37°C, to 8 days, for the samples subjected to the 48-h shift at 37°C. This difference of duration could also explain the more pronounced effect of longer incubations. Indeed, the number of genome copies at the end of the shift to 37°C was not affected by the duration of the exposure (*P* value = 0.126, one-way analysis of variance [ANOVA]), showing that it takes at least 48 h for temperature to affect the number of genome copies. It is therefore possible that the apparent lack of difference between 96 h and 0 h for the 6- and 12-h time points is due to the experiment not being long enough for the effect of temperature to manifest in terms of genome copies. The delay might be due to DNA being slowly released—and degraded by cytosolic DNases—from inactivated bacteria.

### Temperature affects the infectivity of elementary bodies.

Given the effect of temperature on the replication of *R. porcellionis*, we wondered whether it would also affect the infectivity of elementary bodies. To determine this, we incubated elementary bodies of *R. porcellionis* at 20°C (room temperature [RT]) or 37°C and measured the number of IFU every day for 4 days. To be closer to natural conditions, we did not perform the freeze-and-thaw cycle and directly filtered the supernatant from infected Sf9 cells. In addition, as Sf9 cells tend to detach after several days of infection, we used McCoy cells grown at 28°C for IFU quantification of *R. porcellionis* to avoid any bias due to cell detachment. We used a random intercepts mixed-effects linear model to predict the log-transformed count of IFUs based on an interaction variable between the duration of incubation in days and the temperature. When none of the counted cells was infected, we conservatively assumed an IFU count corresponding to one infected cell in 100.

**FIG 5:**
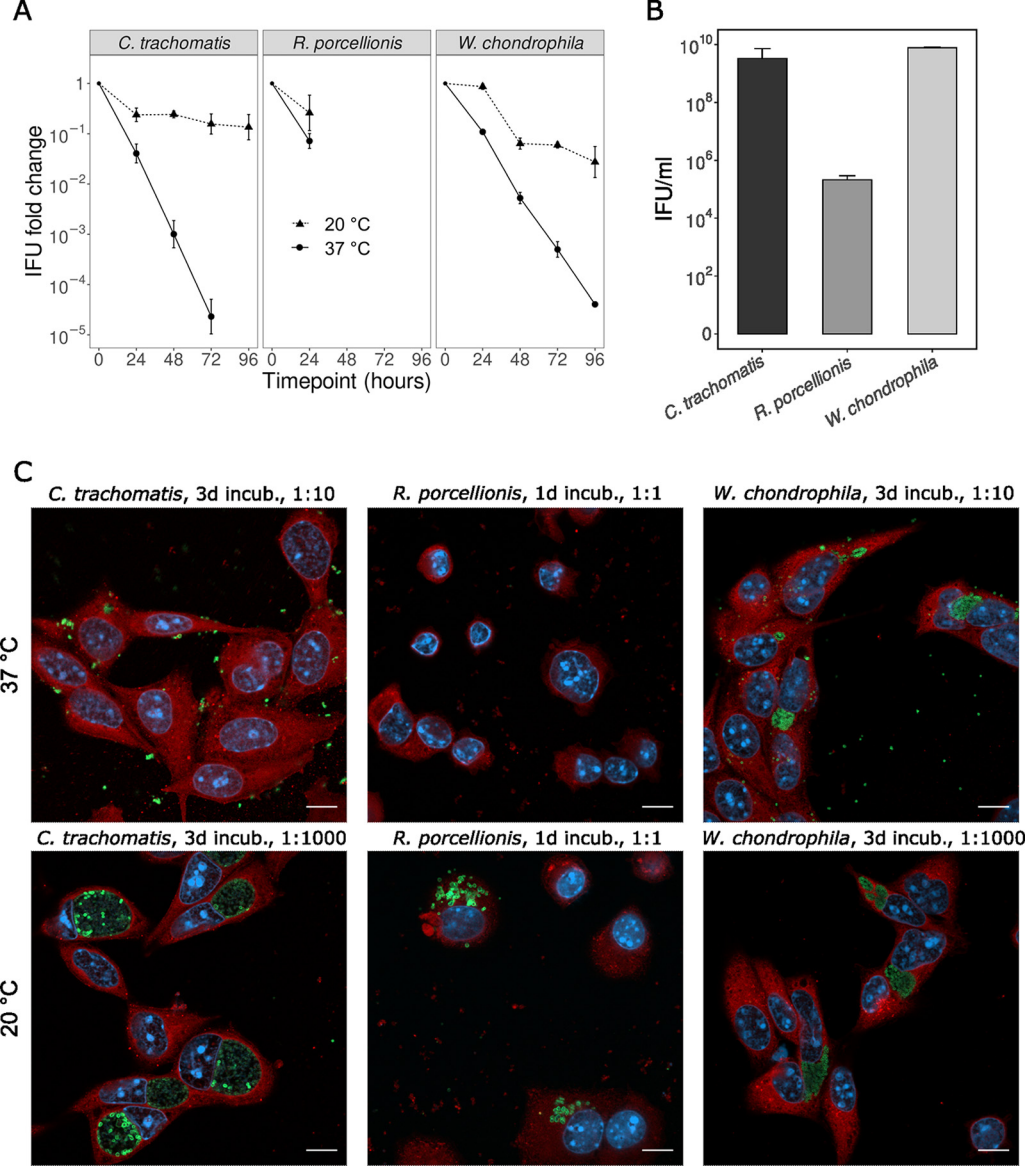
Effect of different incubation temperatures on the infectivity of elementary bodies. (A) Evolution of the number of IFU after incubation at 20°C or 37°C, normalized to the initial IFU count. (B) IFU count at the initial time point. Panels A and B show the results as the mean and standard deviation from three biological replicates. (C) Confocal images of McCoy cells infected with serial dilutions of EBs incubated at 20°C or 37°C. This highlights the deleterious effect of an incubation at 37°C for the EBs of all three species. Interestingly, the inclusions formed by EBs incubated at 37°C also tended to be smaller. The difference between the well-formed inclusions for C. trachomatis and W. chondrophila and the dissemination of reticulate bodies in the host cytoplasm for *R. porcellionis* is also striking. Cells were fixed at 1 day (for C. trachomatis and W. chondrophila) or 6 days (for *R. porcellionis*) postinfection and stained with concanavalin A (red), DAPI (blue), and antibodies against the different bacteria (green). Bar, 10 μm. Incub., incubation duration.

As shown in Fig. 5A, *R. porcellionis* elementary bodies (EBs) incubated for 24 h at 37°C were less infectious than their counterpart incubated at 20°C, although the incubation temperature did not significantly predict the IFU count (*R*^2^ = 0.77, beta for the interaction term = −0.55, *P* value = 0.06). The interaction term implies that for every day of incubation at 37°C, the IFU count will be reduced by a factor of 3.55 (10^0.55^) compared to the same incubation at room temperature. Due to the low initial quantity of infectious particles (Fig. 5B), no infected cell could be observed after the first time point. Using a freeze-and-thaw cycle would only marginally improve the IFU count (Fig. 3D) and introduce an experimental bias, while resorting to bead beating to increase elementary body yield was shown to be counterproductive for *R. porcellionis* (40). In the absence of alternatives to obtain results for later time points, we did not repeat the experiment with these bacteria. We instead resorted to more tractable chlamydiae from two different families, W. chondrophila and C. trachomatis, to check whether exposure to different temperatures also affected their infectivity. As both bacteria infect mammals, we expected their EBs to be adapted to the temperature of their host and the number of IFU to decrease faster at 20°C than at 37°C. This proved to be false, as the IFU count decreased faster at 37°C than at 20°C for both W. chondrophila (*R*^2^ = 0.97, beta = −0.68, *P* value = 3.27 × 10^−11^) and C. trachomatis (*R*^2^ = 0.94, beta = −1.06, *P* value = 2.08 × 10^−10^). Compared to an incubation at room temperature, the IFU count of C. trachomatis and W. chondrophila is thus predicted to be reduced by a factor of 11.48 and 4.79 for every day of incubation at 37°C, respectively. Altogether, these results suggest that while the reticulate bodies of the *Chlamydiae* have different thermal preferences, the elementary bodies of distant families share the same temperature sensitivity.

## DISCUSSION

Several factors are known to influence the host range of the *Chlamydiae.* The ability to inhibit apoptosis has, for instance, been suggested to be a hallmark of chlamydiae infecting multicellular organisms (20, 23), while chlamydiae adapted to multicellular organisms appear to have lost the ability to grow in amoebae (3, 19). The adaptation to specific temperatures has already been demonstrated to be an important determinant for the host range of the members of the *Parachlamydiaceae* family (22, 50, 51). In the present study, we demonstrated that this extends to other families of the *Chlamydiae* phylum by showing that *R. porcellionis* is likely specialized for the temperature ranges encountered in Porcellio scaber (43, 52). As a consequence of this specialization, *R. porcellionis* is quickly inactivated if exposed to the body temperature of mammals, making it unlikely for this bacterium to be pathogenic for mammals and restricting its host range to organisms with a temperature lower than 33°C (Fig. 3A). The use of different incubation temperatures should thus be considered in addition to the inclusion of diverse host cells (40) when attempting to isolate environmental chlamydiae. Indeed, independently of its ability to grow in amoebae, a chlamydial symbiont sharing the same temperature sensitivity as *R. porcellionis* would fail to grow in the Acanthamoeba castellanii subculture system frequently used for the isolation of chlamydiae from environmental samples (6, 12, 53).

The inability to formally exclude that our observations are due to the effect of temperature on the host cells alone is a key limitation of this study. Our experiments, however, make this hypothesis unlikely, as an effect of the temperature exclusively on the host cells would imply the rapid (Fig. 4B) loss of permissivity of Sf9 cells for *R. porcellionis* at 37°C but not for W. chondrophila (Fig. 3A and see Fig. S1 in the supplemental material), as well as the gain of permissivity of mammalian cells at 28°C (Fig. 3B). In addition, a previous work showed that insect cell lines do not lose their permissivity to intracellular bacteria and can notably be used for the culture of *Mycoplasma* species when grown at 37°C (54). Incubations at 37°C also do not seem to affect protein synthesis in Sf9 cells, and heat-tolerant cells growing at 37°C could even be obtained (55). The tolerance of Sf9 cells to high temperatures was hypothesized to be due to Spodoptera frugiperda being adapted to warm climates (55). Along with previous evidence showing a similar temperature sensitivity in species of the *Parachlamydiaceae* family (50, 51), an effect of the temperature on the bacteria themselves thus appears to be the a more parsimonious explanation. Conversely, the effect of cold shock on mammalian cells remains poorly understood and has been studied essentially for its use in the production of recombinant proteins (56, 57). The possibility that the growth of *R. porcellionis* in mammalian cells is due in part to an effect of the temperature on the host cell therefore cannot be excluded.

The attempt to assess whether elementary bodies share the temperature sensitivity of reticulate bodies was impaired by the difficulty of purifying enough *R. porcellionis* EBs. The unequivocal results obtained for two chlamydial species from different families and the trend observed for *R. porcellionis* (Fig. 5A), however, suggest that the elementary bodies of this bacterium also lose their infectivity faster at 37°C. Elementary bodies of different chlamydial species may therefore be more similar in terms of thermal preferences than their respective reticulate bodies (50, 51), although this trend would need to be confirmed in other chlamydiae such as Simkania negevensis or in species of the *Parachlamydiaceae* family. Of note, our results also suggest that the better extracellular survival of Chlamydia-like organisms than of C. trachomatis observed in a previous study (58) may be due to differences in incubation temperatures rather than the metabolic capacity of those organisms.

The ability to grow in both insect and mammalian cells (Fig. 1A and Fig. 3B) that we observed for *R. porcellionis* has also been reported for various Chlamydiaceae (59, 60) species and suggests that despite the long evolutionary distances between those organisms, their cells are still similar enough to allow the growth of bacteria from both families. The long doubling times and frequent aberrant bodies observed in mammalian cells may be due either to differences in the host cell physiology or to the repercussion of a lower fitness of the host cell on the bacteria. In contrast to the previous observation that *R. porcellionis* can grow in different insect cells (40), we could not observe growth in either the C6/36 or the IRE/CTVM19 cell line. The lack of growth in the latter cell line might be due to the specialization of *R. porcellionis* for isopods at the cost of the ability to grow in cells originating from the more distantly related arachnids. This explanation, however, fits poorly with the observation that *R. porcellionis* grows in mammalian cells. The permissivity of cell lines might therefore be more related to the cell type than to the host species, as suggested by the restricted tissue distribution of *R. porcellionis* and Rhabdochlamydia crassificans in their respective hosts (28, 61).

It is unclear how the thermal preferences of *R. porcellionis* generalize to the other members of the *Rhabdochlamydiaceae.* The high predicted diversity of the *Rhabdochlamydiaceae* family (5, 42) and the demonstration that species of the same chlamydial family can have different thermal preferences (50, 51) indeed imply the possibility that other rhabdochlamydiae could have adapted to various ranges of temperatures. However, the similar thermal preferences exhibited by ixodid ticks (44, 62) and woodlice (43, 52) suggest the possibility that the same evolutionary mechanism that drove the adaptation of *R. porcellionis* toward lower temperatures could have had the same effect in tick-borne rhabdochlamydiae (30, 34, 41). This will, however, remain purely speculative until the thermal preferences of additional rhabdochlamydial species can be assessed. Interestingly, a similar temperature sensitivity has also been reported for *Wolbachia* (63, 64), another arthropod symbiont whose host range broadly overlaps that of the *Rhabdochlamydiaceae* (65). Of note, *Wolbachia* appears to influence the thermal preferences of its host to favor its replication (66). A similar effect might also be found in *R. porcellionis*, as it could prevent the accidental cure of the host of the bacterial infection by a short exposure to temperatures higher than 33°C (Fig. 4B).

This work demonstrates the adaptation of *R. porcellionis* to the range of temperatures encountered in its host, at the cost of the ability to successfully infect other species with higher body temperatures. In particular, the temperature sensitivity of *R. porcellionis* precludes its transmission to mammals and excludes a pathogenic role of this bacterium for humans (36–38, 67). It finally highlights the importance of testing different incubation temperatures when attempting to recover chlamydiae from environmental samples.

## MATERIALS AND METHODS

### Cell culture.

Spodoptera frugiperda ovarian epithelial cells (Sf9, ATCC CRL-1711) were cultured at 28°C in Grace insect medium (Gibco, Thermo Fisher Scientific, Waltham, MA, USA) supplemented with 10% fetal calf serum (FCS). Aedes albopictus cells (C6/36, ATCC CRL-1660) were cultured at 28°C in the presence of 5% CO_2_ in Dulbecco modified Eagle medium (DMEM) (PAN-Biotech, Aidenbach, Germany) supplemented with 10% FCS. Ixodes ricinus (IRE/CTVM19) cell lines were maintained at 28°C in Leibovitz L-15 medium (Gibco, Thermo Fisher Scientific, Waltham, MA, USA) supplemented with 10% tryptose phosphate broth (Gibco, Thermo Fisher Scientific, Waltham, MA, USA), 20% FCS, and 1% l-glutamine (Sigma-Aldrich, Buchs, Switzerland), as described in reference 68. Human pneumocytes (A549, ATCC CCL-185), mouse fibroblasts (McCoy, ATCC CRL-1696), and human endometrial cells (Ishikawa, gift of G. Canny) were cultured in DMEM supplemented with 10% FCS and grown at 37°C in the presence of 5% CO_2_. The Acanthamoeba castellanii strain (ATCC 30010) was cultured at 25°C in peptone-yeast extract-glucose (PYG) medium.

### Bacterial strains.

The *R. porcellionis* strain was acquired from the DSMZ collection (DSM 27522) and cultivated in Sf9 cells. The infected cells were passaged once a week, and fresh cells were added approximately every four passages to compensate for host cell death due to the presence of the bacteria. Waddlia chondrophila strain WSU 86-1044 (ATCC VR-1470) was cocultivated with A. castellanii in PYG broth at 32°C. Suspensions of EBs were collected at 7 days postinfection, diluted 10 times, and used to infect fresh A. castellanii. Chlamydia trachomatis (ATCC VR-902B) was cultivated in McCoy cells incubated at 37°C in DMEM supplemented with 10% FCS and 1 μg mL^−1^ cycloheximide.

### Infection procedure.

Cells were seeded in a 24-well plate (Corning) at a density of 1 × 10^5^ or 3 × 10^5^ cells per well 2 h before infection. The infection procedures were performed as described in previous publications (26, 40).

For *R. porcellionis*, suspensions of infected Sf9 cells were subjected to a freeze-thaw cycle to disrupt the cells, followed by a filtration through a 5-μm-pore filter to remove the debris. Plated cells were then infected with the filtrate at a multiplicity of infection (MOI) of ~0.1 to 1 and centrifuged for 15 min at 130 × *g* at room temperature, followed by an incubation of 30 min at 28°C. The medium was then replaced to remove noninternalized bacteria.

W. chondrophila EBs were collected from the supernatant of A. castellanii at 5 days postinfection. The supernatant was then filtered through a 5-μm-pore filter to remove cell debris. Plated cells were infected with the filtrate at an MOI of ~0.1 to 1 and centrifuged at 1,790 × *g* for 10 min at room temperature. After 30 min of incubation at either 28°C (for Sf9 cells) or 37°C (for mammalian cells), the medium was replaced to remove noninternalized bacteria.

C. trachomatis EBs were collected from the supernatant of infected McCoy cells at 3 days postinfection. The supernatant was then filtered with a 5-μm-pore filter. Plated cells were infected with the filtrate and centrifuged at 900 × *g* for 15 min at room temperature. The infected cells were then incubated for 30 min at 37°C, and the medium was replaced to remove noninternalized bacteria.

After the infection, the cells were incubated at their usual growth temperature, unless specified otherwise. The samples were collected at various time points for quantification by quantitative PCR (qPCR) and immunofluorescence staining. The MOI was estimated by measuring the proportion of infected cells with 10-fold serial dilutions of EB filtrate in immunofluorescence.

### Inclusion-forming unit (IFU) quantification.

Cells were plated in a 24-well plate at a density of 3 × 10^5^ cells per well 2 h before the infection and were then infected with serial 10-fold dilutions of EB suspensions. After the initial 30 min of incubation, the medium was replaced with fresh medium supplemented with 1 μg mL^−1^ of cycloheximide (69). The cells infected with W. chondrophila or C. trachomatis were incubated for 24 h at 37°C with 5% CO_2_, while the cells infected with *R. porcellionis* were incubated for 6 days at 28°C, with 5% CO_2_ for mammalian cells. The cells were then fixed and stained for immunofluorescence, and the proportion of infected cells was determined using an epifluorescence microscope. At least 100 cells were counted for each condition.

### Effect of temperature on EB infectivity.

C. trachomatis and W. chondrophila EBs were collected from the supernatant of infected cells as in a standard infection procedure. To be closer to a natural infection, EBs of *R. porcellionis* were collected from the supernatant of infected cells, without any prior lysis step. The supernatants were filtered with a 5-μm-pore filter. The filtrate was then diluted 1:1 in PYG for W. chondrophila, 1:1 in DMEM supplemented with 10% FCS for C. trachomatis, and 1:1 in Grace medium supplemented with 10% FCS for *R. porcellionis*. The suspensions of elementary bodies were then incubated at either 20°C or 37°C in 24-well plates. In the case of C. trachomatis, the plate was incubated in the presence of 5% CO_2_. IFUs were quantified using McCoy cells immediately after the dilution in fresh medium or after 1, 2, 3, or 4 days of incubation.

### Effect of incubation temperature on growth.

Sf9 cells were plated in a 24-well plate at a density of 10^5^ cells per well, infected with *R. porcellionis* at an MOI of ~0.1 to 1, and incubated for 48 h at 28°C. The plates were then incubated at 37°C for 6, 12, 24, or 48 h before being switched back to 28°C for four additional days. Samples were taken for bacterial growth quantification by qPCR right after the switch to 28°C and after the subsequent 4 days of incubation.

### Quantitative PCR.

Genomic DNA was extracted using the Wizard SV genomic DNA purification kit (Promega, Dübendorf, Switzerland) following the manufacturer’s protocol. Quantitative PCR for *R. porcellionis* (36) or W. chondrophila (70) was performed on 5 μL of genomic DNA with iTaq Supermix (Bio-Rad, Cressier, Switzerland), 200 nM primers (WadF4, 5′-GGCCCTTGGGTCGTAAAGTTCT-3′, and WadR4, 5′-CGGAGTTAGCCGGTGCTTCT-3′, for W. chondrophila; RcF, 5′-GACGCTGCGTGAGTGATGA-3′, and RcR, 5′-CCGGTGCTTCTTTACGCAGTA-3′, for *R. porcellionis*) and 100 nM probe (WadS2, 5′-6-carboxyfluorescein [FAM]-CATGGGAACAAGAGAAGGATG-BHQ1-3′, and RcS, 5′-FAM-CTTTCGGGTTGTAAAACTCTTTCGCGCA-BHQ1-3′). The cycling conditions were identical for both qPCRs: 3 min at 95°C and 40 cycles of 15 s at 95°C and 1 min at 60°C. The qPCRs were performed on a QuantStudio3 real-time PCR system (Applied Biosystems, Thermo Fisher Scientific, Waltham, MA, USA).

### Immunofluorescence staining.

Infected cells grown on glass coverslips were fixed with ice-cold methanol for 5 min at different time points after the infection. Cells were then washed three times with phosphate-buffered saline (PBS) and incubated for at least 2 h in PBS with 0.1% saponin, 0.04% NaN_3_, and 10% FCS (blocking solution). The coverslips were then incubated at room temperature for 2 h in blocking solution with rabbit anti-Simkania negevensis antibodies (25) (dilution at 1:1,000), rabbit anti-Waddlia chondrophila antibodies (71) (dilution at 1:1,000), or goat antibodies targeting the major outer membrane protein of Chlamydia trachomatis (dilution at 1:1,000) (LSBio, Seattle, WA, USA). Anti-*Simkania* antibodies were used to detect *R. porcellionis*, as antibodies raised against a chlamydial species often cross-react with related species (40, 72). After the incubation with the primary antibody, the coverslips were washed three times in PBS with 0.1% saponin and incubated for 1 h at room temperature in blocking solution with 1.6 μg mL^−1^ 4′,6-diamidino-2-phenylindole (DAPI) dilactate (Molecular Probes, Thermo Fisher Scientific, Waltham, MA, USA), 100 μg mL^−1^ concanavalin A-Texas Red conjugate (Invitrogen, Thermo Fisher Scientific, Waltham, MA, USA), and Alexa 488-conjugated chicken anti-goat or goat anti-rabbit antibodies (1:1,000 dilution) (Life Technologies, Thermo Fisher Scientific, Waltham, MA, USA). The coverslips were then embedded in Mowiol (Sigma-Aldrich, Buchs, Switzerland) and kept in the dark at 4°C until further use. The coverslips were examined with a confocal microscope (Zeiss LSM 900; Zeiss, Oberkochen, Germany).

### Electron microscopy imaging.

Sf9 and McCoy cells were plated in T25 flasks at a density of 10^6^ cells per flask and infected with *R. porcellionis* at an MOI of ~1. After the initial 30 min of incubation at 28°C, the old medium was replaced with fresh medium supplemented with 1 μg mL^−1^ cycloheximide. The cells were then incubated for 6 days at 28°C before collection. The cell suspension was centrifuged at 500 × *g* for 10 min, and the pellet was resuspended in a solution of 4% paraformaldehyde (Electron Microscopy Sciences [EMS], Hatfield, PA, USA) and 2.5% glutaraldehyde (Fluka, Buchs, Switzerland) in a 0.1 mol L^−1^ phosphate buffer at pH 7.4 (PB buffer) and incubated at 4°C for 4 h. After an additional centrifugation at 500 × *g* for 10 min, the cells were resuspended in a solution of 1% paraformaldehyde in PB buffer. They were then directly postfixed by a fresh mixture of 1% osmium tetroxide (EMS, Hatfield, PA, USA) with 1.5% potassium ferrocyanide (Sigma, St. Louis, MO, USA) in PB buffer for 1 h at room temperature (RT). The samples were then washed three times in distilled water and spun down in 2% low-melting-temperature agarose in H_2_O (Sigma, St. Louis, MO, USA), allowed to solidify on ice, cut in 1-mm^3^ cubes, and dehydrated in acetone solution (Sigma, St. Louis, MO, USA) at graded concentrations (30%, 40 min; 50%, 40 min; 70%, 40 min; 100%, twice for 1 h). This was followed by infiltration in Epon (Sigma, St. Louis, MO, USA) at graded concentrations (Epon 1/3 acetone, 2 h; Epon 3/1 acetone, 2 h, Epon 1/1, 4 h; Epon 1/1, 12 h) and finally polymerization for 48 h at 60°C in an oven. Ultrathin sections of 50 nm were cut on a Leica Ultracut microtome (Leica Microsystems GmbH, Vienna, Austria) and picked up on a copper slot grid (2 by 1 mm; EMS, Hatfield, PA, USA) coated with a polyethyleneimine (PEI) film (Sigma, St. Louis, MO, USA). Sections were poststained with 2% uranyl acetate (Sigma, St. Louis, MO, USA) in H_2_O for 10 min, rinsed several times with H_2_O followed by Reynolds lead citrate in H_2_O (Sigma, St. Louis, MO, USA) for 10 min, and rinsed several times with H_2_O. Images were taken with a Philips CM100 1201 microscope at the Lausanne University electron microscopy facility.

### Statistical analysis.

The results of this study are given as means with standard deviations. The linear regression models with random effect were fitted using the lme4 package (73). Doubling times were calculated by dividing 48 h (the interval between time points used in this work) by the log_2_ of the highest fold change observed between two consecutive time points. All statistics were performed with R (v4.2.0).
