# Supplementary material for: Temperature Affects the Host Range of Rhabdochlamydia porcellionis
Source: Appl Environ Microbiol. 2023 Apr 12;89(5):e00309-23. doi: 10.1128/aem.00309-23 (PMC10231146; doi:10.1128/aem.00309-23)

Supplementary figure 1. Growth kinetics of *Waddlia chondrophila* in Sf9 cells at 37 or 28 °C. *W. chondrophila* was chosen as a control for its ability to grow at both temperatures. The results suggest that Sf9 do not lose their permissivity to chlamydiae at 37 °C. Data are represented as the mean and standard deviation of three biological replicates.

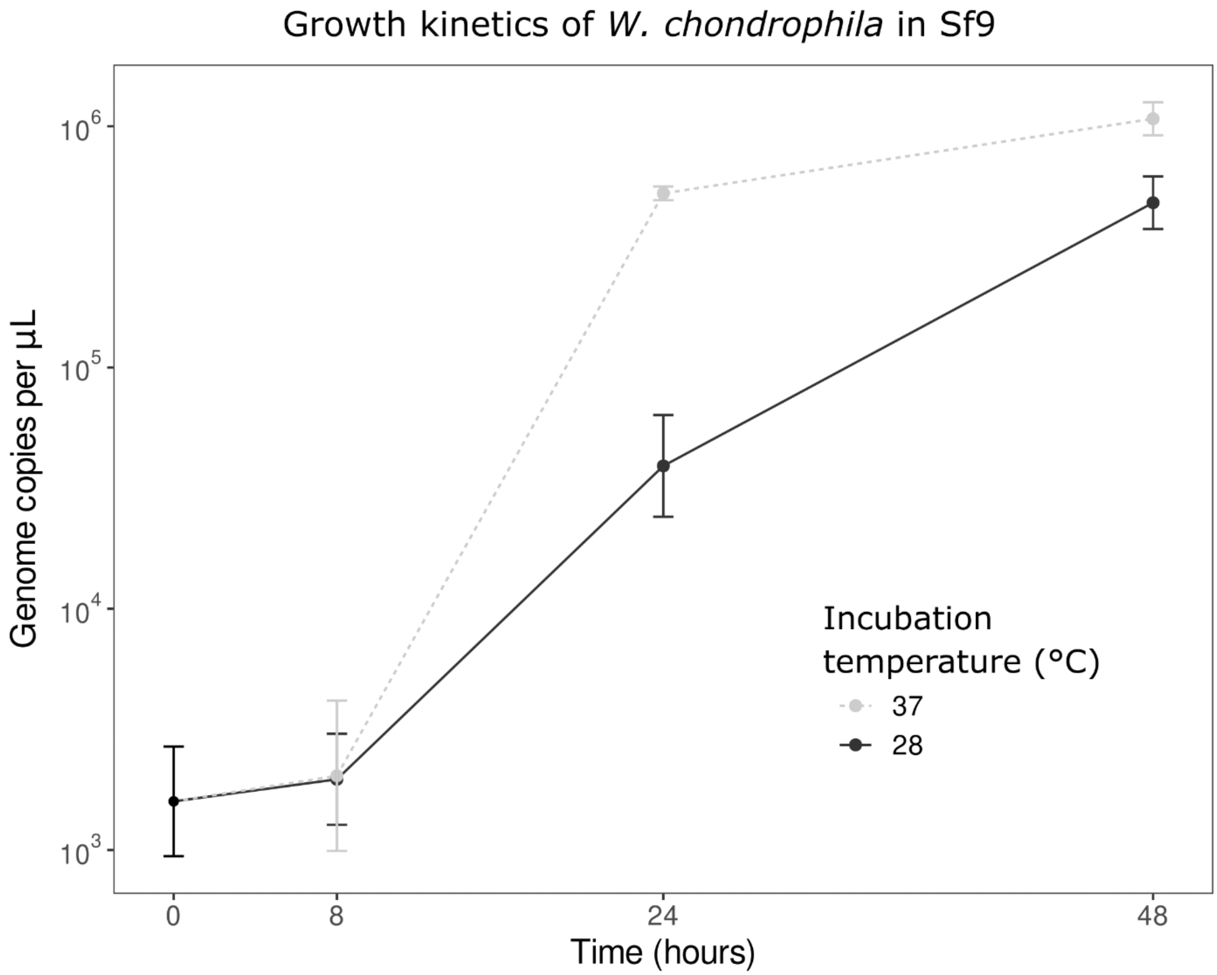

Supplementary figure 2. TEM micrographs of *R. porcellionis* in McCoy cells at 6 days post-infection. Several bacteria have a concave shape, contrasting to the appearance of bacteria in Supplementary Fig. 1. As the preparation method was identical as for Sf9 cells, it is unlikely to be a fixation artifact. No intermediate body can be observed. Scale bar: 1 µm (A), 500 nm (B) or 200 nm (C).

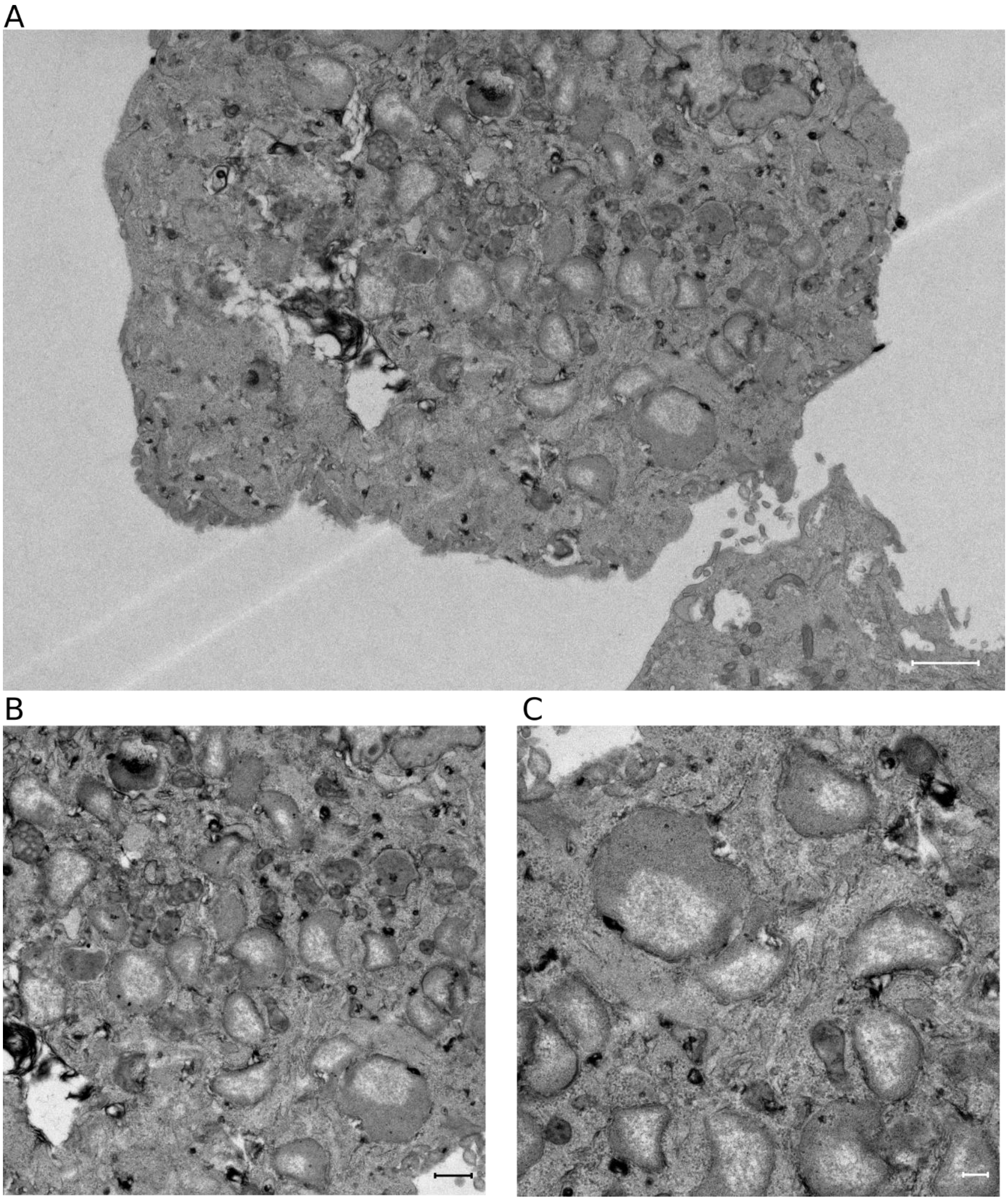

Supplement: Supplemental file 1 — Supplemental material. Download aem.00309-23-s0001.pdf, PDF file, 1.0 MB [file aem.00309-23-s0001.pdf]
